# Unveiling the Physicochemical Properties of Magnetic Nanoparticles as Solid Carriers for Laccase Immobilization Toward Different Reducing Substrates

**DOI:** 10.3390/biom16091244

**Published:** 2026-08-27

**Authors:** Jessica Costa, Andrea Atrei, Juan José Valle-Delgado, Monika Österberg, Rebecca Pogni

**Affiliations:** 1 Department of Biotechnology, Chemistry and Pharmacy, University of Siena, Via Aldo Moro 2, 53100 Siena, Italy; jessica.costa2@unisi.it (J.C.); andrea.atrei@unisi.it (A.A.); 2 Department of Bioproducts and Biosystems, Aalto University, Vuorimiehentie 1, 02150 Espoo, Finland; juanjose.valledelgado@aalto.fi (J.J.V.-D.); monika.osterberg@aalto.fi (M.Ö.)

**Keywords:** laccase, enzymatic immobilization, magnetic nanoparticles, reducing substrates

## Abstract

Laccases are multicopper oxidases capable of oxidizing a wide range of substrates using molecular oxygen as the terminal electron acceptor, producing water as the sole by-product. High-redox potential fungal laccases, such as those from *Trametes versicolor*, are particularly attractive for industrial and environmental applications, although their use is often limited by sensitivity to operational conditions. Enzyme immobilization represents an effective strategy to enhance laccase stability and reusability. In this work, magnetic nanoparticles (MNPs) were investigated as support for laccase immobilization due to their high surface area, biocompatibility, and ease of magnetic recovery. Two modified co-precipitation synthetic routes were systematically evaluated, and the size, morphology, and chemical composition of the products were characterized by microscopy, light scattering, and spectroscopic methods, while both adsorption and covalent immobilization strategies were explored. The MNP surface was found to be highly reactive toward radical species generated during laccase-catalyzed reactions, especially in the presence of small Fe^2+^ excess. While this can enhance the enzyme catalytic activity, it challenges the inertness of the support and promotes, in some cases, strong interactions between reaction products and the nanoparticle surface. These findings highlight a previously unexplored role of magnetic supports in laccase-based biocatalytic systems.

## 1. Introduction

Laccases (EC 1.10.3.2) are multicopper oxidases widely distributed in plants, fungi, bacteria, and insects, and they have attracted considerable interest due to their ability to catalyze the oxidation of a broad range of substrates using molecular oxygen as the terminal electron acceptor, producing water as a by-product. Their catalytic activity is governed by a copper-containing active site composed of four copper ions (T1, T2, and T3), whose redox properties strongly influence substrate specificity and reaction efficiency. Based on the redox potential of the T1 copper site, laccases are commonly classified as low-, medium-, or high-redox potential enzymes. Among them, fungal laccases, particularly those from wood-decaying basidiomycetes, such as *Trametes versicolor*, exhibit the highest redox potentials and the broadest substrate range [1]. Like other enzymes, laccases are highly efficient biocatalysts with applications spanning multiple sectors, representing a significant advancement in industrial biotechnology [2]. They originate from renewable resources, are GRAS (Generally Recognized As Safe) enzymes, operate under relatively mild temperature and pH conditions, and display high substrate selectivity [3,4]. However, despite their catalytic versatility, laccases are highly sensitive to environmental conditions. Their activity can be significantly affected by substrate complexity, inhibitory compounds, and non-optimal operational conditions, while certain metal ions have been reported to enhance enzyme stability and catalytic efficiency [5]. To mitigate this pronounced environmental sensitivity and improve operational robustness, enzyme immobilization has emerged as an effective strategy to enhance laccase stability, limit denaturation, and preserve catalytic activity, thereby expanding its industrial and environmental applicability [6,7,8]. In this context, the choice of support material plays a crucial role. Ideal supports should exhibit high thermal and chemical stability, inertness under reaction conditions, strong affinity for enzymes, reusability, low cost, suitable surface functional groups, and good biocompatibility [9].

Advances in nanotechnology have further expanded immobilization strategies, with magnetic nanoparticles (MNPs) attracting particular attention due to their magnetic responsiveness, low toxicity, and biocompatibility [10,11]. Magnetic nanoparticles have been extensively studied and successfully applied for the immobilization of several classes of enzymes, including hydrolytic enzymes [12,13] and oxidative enzymes, such as laccase [14]. However, when considering redox enzymes such as laccases and supports containing Fe^2+^/Fe^3+^ redox-active components, special attention must be paid to the true inertness of the support. The redox processes associated with laccase catalysis may interfere with the nanoparticle surface, leading to surface modification or strong adsorption of reaction products [15]. While such interactions may, on one hand, enhance laccase catalytic activity through synergistic redox effects, they can, on the other hand, compromise the inert nature of the support and hinder product recovery due to adsorption onto the nanoparticle surface.

Within this framework, the present study aims to elucidate the role of magnetic nanoparticles in laccase immobilization and reaction towards different reducing substrates. Two widely used and optimized co-precipitation-based synthetic procedures were compared, focusing on the key parameters differentiating the methods and, in particular, on their impact on enzyme immobilization performance. The MNPs, as reported in the literature, as magnetite-based catalysts in oxidation reactions, offer key advantages, including facile magnetic separation and catalyst reusability. Despite some limitations, their advantages make magnetic nanocatalysts attractive for catalytic and environmental applications [16].

Nonetheless, the surface of the nanoparticles was found to be highly susceptible to attack by radical species generated during laccase-catalyzed reactions, especially when a small excess of Fe^2+^ salt is used in the preparation of magnetite MNPs. Consequently, from an immobilization standpoint, the reaction products are not always clearly detectable in solution, as they may strongly interact with or bind to the nanoparticle surface.

In this context, different laccase substrates were investigated using laccase immobilized on MNPs, revealing the formation of radical species and their subsequent interaction with the magnetic support. The interaction between radical species generated during laccase-catalyzed reactions and magnetic supports may represent an additional advantage for the functional coating from one side but, at the same time, can hamper the use of this support for some reducing substrates.

## 2. Materials and Methods

### 2.1. Materials

Iron (III) chloride hexahydrate (FeCl_3_·6H_2_O), ammonium iron (II) sulfate hexahydrate (Fe(NH_4_)_2_ (SO_4_)_2_·6H_2_O), iron (II) chloride (FeCl_2_), iron (III) chloride (FeCl_3_), (3-aminopropyl) triethoxysilane (APTES), glutaraldehyde (25% aqueous solution), laccase from *T. versicolor* (U/mg ≥ 0.5), 2,2′-azino-bis(3-ethylbenzothiazoline-6-sulfonic acid) (ABTS), 3-amino-4-hydroxybenzene sulfonic acid, and dopamine hydrochloride were obtained from Sigma–Aldrich (St. Louis, MO, USA). Sodium hydroxide (25% *w*/*w*), ethanol, and hydrochloric acid were obtained from Merck (Darmstadt, Germany). Phosphate buffer (100 mM) at pH 7.1 and acetate buffer (100 mM) at pH 4.5 were used for the experiments.

### 2.2. Methods

#### 2.2.1. Synthesis of MNPs

MNPs were prepared by two co-precipitation methods. MNPs (indicated hereafter as MNPs-1) were prepared by co-precipitation from solutions of FeCl_3_·6H_2_O and Fe(NH_4_)_2_(SO_4_)_2_·6H_2_O (Mohr salt) in double-distilled water (DDW) by adding an appropriate volume of 1 M NaOH. A total of 10% excess of Mohr salt with respect to the stoichiometric Fe^2+^/Fe^3+^ ratio 1:2 was used to compensate for possible oxidation of Fe^2+^. The reaction was carried out at 60 °C, and the solution was deoxygenated by N_2_ bubbling. The black precipitate of MNPs was separated from the solution by means of a magnet, washed several times with DDW, and dried in a flux of N_2_ [17,18].

In the second preparation procedure, magnetite MNPs (indicated in the following as MNPs-2) were prepared by co-precipitation from solutions of FeCl_3_·6H_2_O (1.62 g, 10 mM) and FeCl_2_ (0.64 g, 5 mM) (stoichiometric Fe^2+^/Fe^3+^ ratio 1:2) in DDW (50 mL) by adding 3 mL of ammonia solution (conc. 25 wt %) drop by drop. The reaction was carried out for 1 h at room temperature under N_2_ atmosphere. MNPs-2 were separated from the solution by cycles of ultracentrifugation (30,000 rpm, 1 h at 20 °C) and washed with DDW. At the end, MNPs-2 were freeze-dried and collected as powder. In Table 1, the two synthetic routes are summarized [19].

#### 2.2.2. MNP Characterization

The size and morphology of MNPs were analyzed by means of Atomic Force Microscopy (AFM) and Transmission Electron Microscopy (TEM). MNPs-1 were characterized with AFM using a Solver Pro microscope (NT-MDT, Moscow, Russia). AFM images were acquired in semi-contact mode using a Si cantilever with a force constant of 5 N/m working at a frequency of ca. 150 kHz. Samples for the AFM measurements were prepared by depositing drops of diluted dispersions of MNPs on glass coverslips.

Concerning MNPs-2, a drop of MNPs suspension (100 mg/mL) was deposited on a silica surface and left overnight at room temperature. Subsequently, the silica surface was gently rinsed with water and dried with nitrogen. The sample was imaged in tapping mode in air using a MultiMode 8 AFM equipped with a J scanner and a Nanoscope V controller from Bruker Corporation (Billerica, MA, USA). An NCHV-A probe from Bruker, a scan rate of 0.996 Hz, a tip velocity of 9.96 μm/s, and a scan size of 5 μm × 5 μm were used for the imaging. Furthermore, MNP-2 nanoparticles were also characterized by TEM. An FEI Tecnai 12 (Hillsboro, OR, USA) instrument operating at 120 kV was used.

The surface composition of MNPs-1 and MNPs-2 was analyzed by Fourier-Transform Infrared Spectroscopy (FT-IR) using a Bio-Rad FTS 6000 Spectrometer (Bio-Rad, Hercules, CA, USA) equipped with a Ge crystal. Spectra were acquired in attenuated total reflection mode in the wavenumber range of 600–4000 cm^−1^ at room temperature with a resolution of 4 cm^−1^.

The pH stability was evaluated by preparing six samples of MNPs-1 and MNPs-2 at pH values ranging from 4 to 9. The same amount of MNPs was suspended in distilled water, and then the pH was adjusted using NaOH 0.1 M and HCl 0.1 M to have a final volume of 5 mL. The size distribution and ζ-potential of magnetic nanoparticle samples were analyzed by dynamic light scattering using a Zetasizer NanoZS90 instrument (Malvern, Worcestershire, UK). The ζ-potential was determined with a dip cell probe. Three replicates of the measurements were carried out.

#### 2.2.3. Immobilization Procedures

Laccase from *Trametes versicolor* (*Tv*) was immobilized on magnetic nanoparticles using the physical absorption and covalent methods.

- Physical Adsorption on MNPs-1 and MNPs-2

Laccase solution was left to be adsorbed directly onto the MNPs suspension and left to react at 25 °C in a shaker (150 rpm) for 16 h. After that, the magnetic nanoparticles with laccase were separated from the suspension using a magnet and washed one time with 1 mL of acetate buffer pH 4.5, 100 mM. The washing buffer was collected to test the activity of the unbound enzyme.

- Covalent immobilization on MNPs-1

The MNPs-1 were modified by APTES to introduce the amino group on Fe_3_O_4_ MNPs. MNPs-1 (1 g, 4.32 · 10^−3^ mol) were suspended in EtOH (332 mL). Then, APTES (8.0 mL, 0.034 mol) was added to the MNP suspension, and the pH = 5 was adjusted by the drop-wise addition of HCl 1N. After stirring at 25 °C for 24 h, the amino-coated Fe_3_O_4_ nanoparticles were collected using a permanent magnet, washed with ethanol three times, and then with distilled water and dried under N_2_ flux. The amino-coated Fe_3_O_4_ nanoparticles were suspended in phosphate buffer (PBS) (100 mM, pH 7.1) and sonicated for 15 min. Later, the glutaraldehyde solution was added to the suspension, which was maintained under stirring at 150 rpm at 25 °C for 2 h. The samples of MNPs-1-APTES-glutaraldehyde were collected using a permanent magnet and washed with phosphate buffer three times to remove the unreacted glutaraldehyde [20]. The laccase solution was added to MNPs-1-APTES–glutaraldehyde complexes, and the suspension was stirred at 150 rpm at 25 °C for 16 h. Finally, the magnetic nanoparticles Fe_3_O_4_–laccase were separated magnetically and washed two times with 0.5 mL of acetate buffer (100 mM, pH 4.5) to remove the free enzyme. The washing buffer was collected for the determination of the possible remaining activity.

#### 2.2.4. Catalytic Activity Assays of Immobilized Laccase

The activity of the immobilized laccase was measured using 3-amino-4-hydroxybenzene sulfonic acid (0.2 mM) as a substrate in acetate buffer (pH 4.5). The formation of a colored compound (2-amino-3-oxo-3H-phenoxazine-8-sulfonic acid) was monitored by measuring the absorbance increase at 420 nm (ε/dm^3^ mol^−1^ cm^−1^ 8600) with a UV-Vis spectrophotometer from Lambda 900/Perkin Elmer Instruments (Norwalk, CT, USA) [21]. One unit of activity is defined as the amount of enzyme required to oxidize 1 μmol of substrate per minute.

The immobilization process was optimized by changing the amount of support (25, 50, 100 mg of nanoparticles) and the concentration of laccase (0.5, 1, 2 mg/mL of laccase). The best conditions, 50 mg of support and 0.5 mg/mL of laccase, were used for all experiments. The immobilization yield is used to describe the percentage of total enzyme activity from the free enzyme solution that is immobilized:

(1) $$Immobilization\;yied\;\left(\%\right)=\frac{\left(A_{f}-A_{wb}\right)}{A_{f}}\times 100,$$

where A_f_ is the free laccase activity and A_wb_ corresponds to the laccase activity in washing buffer after the immobilization process.

With activity recovery, the activity of the immobilized enzyme is compared to that of the total starting activity of the free enzyme [6]:

(2) $$Activity\;recovery\;\left(\%\right)=\frac{A_{im}}{A_{f}}\times 100$$

where A_im_ is the activity of the immobilized enzyme and A_f_ is the activity of the free enzyme.

#### 2.2.5. Catalytic Reaction with Different Reducing Substrates

Three different reducing substrates for laccase, 2,2′-azino-bis (3-ethylbenthiazoline-6-sulfonic acid) (ABTS), 3-amino-4-hydroxybenzene sulfonic acid, and 3,4-dihydroxyphenethylamine (dopamine), were used to test the free and immobilized enzyme. Free laccase solution was added to the substrate solution in acetate buffer at pH 4.5 (final concentration: free laccase 0.002 mM/substrate 0.2 mM—molar ratio laccase:substrate = 1:100). After 30 min, the solution was analyzed using a UV-Vis spectrophotometer from Lambda 900/Perkin Elmer Instruments (Norwalk, CT, USA). The same procedure was carried out for laccase-MNPs-1 and laccase-MNPs-2. Substrate solution was added to magnetic nanoparticles with immobilized laccase (laccase substrate 1:100 molar ratio), and then the solution was separated using a magnet and analyzed.

For ABTS, the reaction with free and immobilized laccase was monitored over time, including at t0, when the reaction is started, and after 5 min (t1), 10 min (t2), 20 min (t3), and 30 min (t4). The reaction was followed by monitoring the formation of the ABTS cation radical at 420 nm (ε = 3.6 × 104 M^−1^ cm^−1^) using the UV-Vis technique. To monitor the reaction with the substrates and the supported enzyme on MNPs, 50 mg of MNPs-1 and MNPs-2 were dispersed in 0.2 mM ABTS solution (in acetate buffer, with pH 4.5 and 100 mM). The reaction mix was left to react for 30 min, magnetic nanoparticles were separated using a magnet, and the solution was analyzed by UV-Vis.

The same procedure was used to test the substrate 3-amino-4-hydroxybenzene sulfonic acid, and the formation of phenoxazine dye was monitored by measuring the absorbance increase at 420 nm (ε = 8600 × 10^4^ M^−1^ cm^−1^).

Laccase activity was also tested with dopamine, using the same protocol as the other substrates. The reaction mix was left to react for 1 day, and the solution was analyzed by UV-Vis. The possible presence of the substrate on the MNP surface was tested by FT-IR analysis. The samples were prepared under the same conditions, but with increasing substrate concentration (molar ratio—laccase: substrate—1:10). The spectra were recorded before and after the reaction with the substrates. The samples were dried by nitrogen flux before the analysis.

## 3. Results and Discussion

### 3.1. MNP Physicochemical Characterization

**Figure 1:**
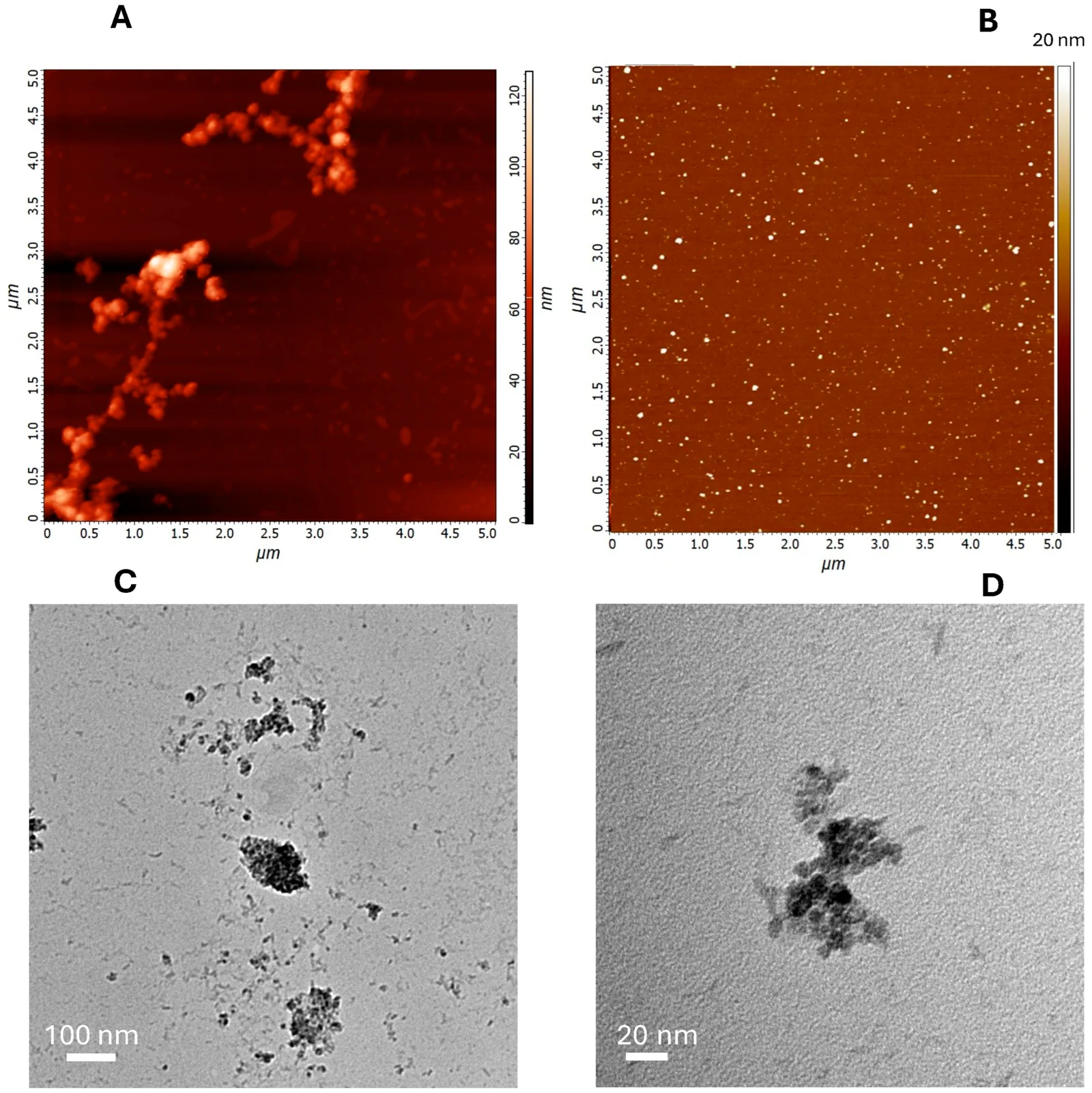
Morphological characterization of bare MNPs-1 and MNPs-2. (**A**) AFM of MNPs-1; (**B**) AFM of MNPs-2; both scans are 5 μm × 5 μm; (**C**) TEM of MNPs-2 (scale bar: 100 nm); (**D**) TEM of MNPs-2 (scale bar: 20 nm).

**Figure 2:**
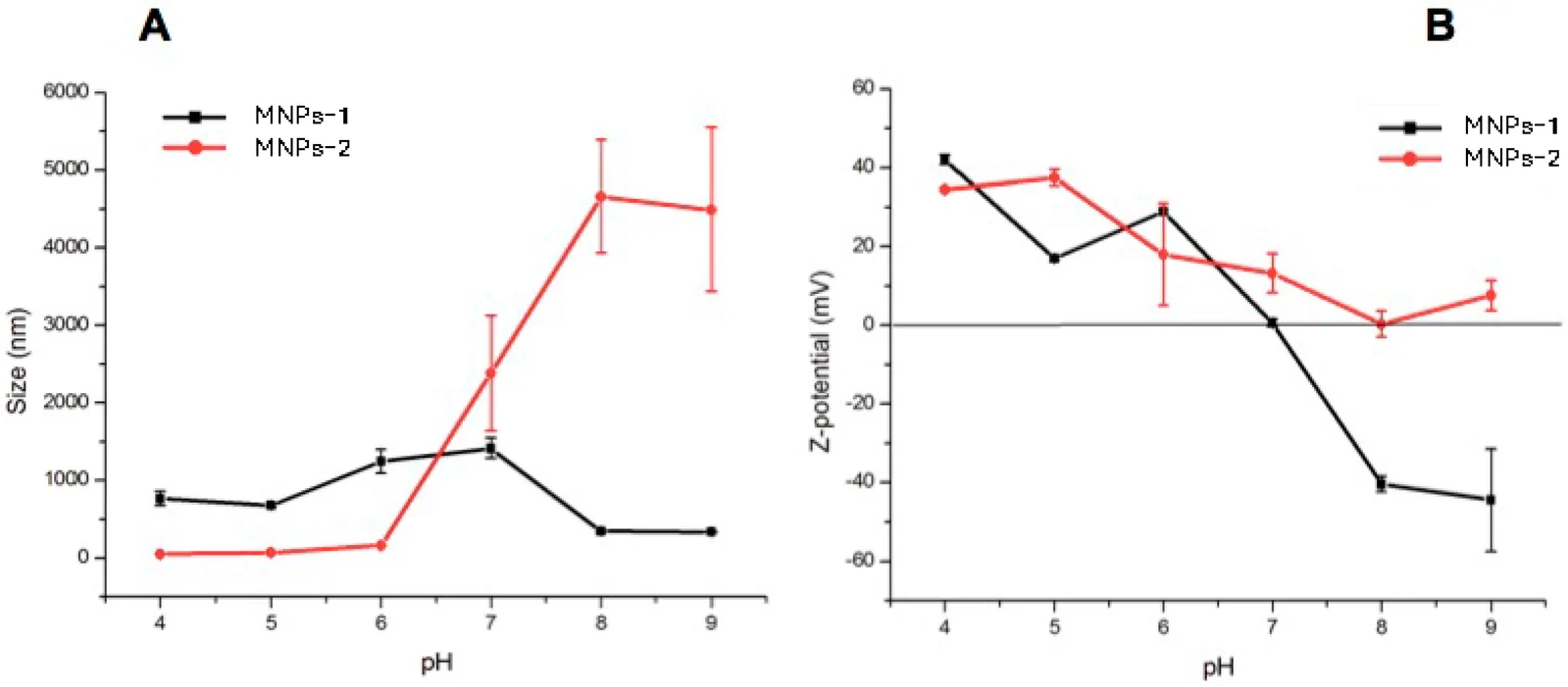
pH-dependent stability of MNPs: (**A**) hydrodynamic size distribution of MNPs-1 and MNPs-2 as a function of pH (4–9); (**B**) ζ-potential profiles of MNPs-1 and MNPs-2 measured across the same pH range.

For both MNPs-1 and MNPs-2, the dimensions of individual nanoparticles and their aggregates were evaluated using AFM. Comparison of the images (Figure 1A,B) shows that MNPs-1 tend to form relatively large aggregates, whereas MNPs-2 are more uniformly dispersed, and individual MNPs are visible. Within some MNP-1 clusters, individual nanoparticles can be distinguished, with estimated sizes (from height measurements) of approximately 20–25 nm. The significantly smaller size of MNPs-2 is further confirmed by TEM analysis (Figure 1C,D), where individual nanoparticles range from 2 to 10 nm. The differences observed in the size of individual MNPs and their aggregates can be attributed to the distinct preparation conditions. Parameters such as temperature, ionic strength, and the presence or absence of an inert atmosphere significantly influence nanoparticle aggregation [22,23,24]. In particular, the preparation conditions adopted for MNPs-1 result in reduced electrostatic repulsion between nanoparticles, thereby promoting the formation of larger aggregates compared to MNPs-2. This behavior is consistent with AFM and TEM observations (Figure 1), as well as with DLS measurements (Figure 2). Among all the parameters investigated, the excess of Fe^2+^ was identified as a key factor significantly affecting the inertness of the immobilization support. Magnetite nanoparticles are typically synthesized using a Fe^2+^/Fe^3+^ molar ratio of 1:2; however, in some cases, a slight excess of Fe^2+^ (e.g., Fe^2+^/Fe^3+^ = 1.1:2 as for MNPs-1) is introduced to compensate for possible Fe^2+^ oxidation during synthesis and to maintain the desired stoichiometric ratio [25,26,27]. As demonstrated in this study, even a small excess of Fe^2+^ used in the preparation can influence the inertness of the magnetic support, particularly in oxidative processes catalyzed by laccase, thereby promoting interactions between the nanoparticle surface and reactive radical species. In our previous works, MNPs-1, prepared with a small excess of Fe^2+^, were characterized by XRD and XPS [17,18]. The diffraction peaks of iron oxyhydroxide (FeOOH) or hematite phase were not detected in the diffractograms. However, with XRD, it is very difficult to distinguish between Fe_3_O_4_ (magnetite) and γ-Fe_2_O_3_ (maghemite) since they have the same structure and very similar lattice parameters. The analysis of these nanoparticles by XPS shows, consistently with results reported in the literature, that it is not straightforward to discriminate between Fe^2+^ and Fe^3+^ and to determine the stoichiometry at the surface of the nanoparticles [18].

To evaluate the colloidal stability of MNPs-1 and MNPs-2 dispersions, a series of aqueous dispersions with pH values ranging from 3 to 9 were prepared, and their hydrodynamic diameter and ζ-potential were measured (Figure 2). The surface charge of magnetic nanoparticles is strongly pH-dependent. In aqueous media, the nanoparticle surface is covered by –FeOH groups. At acidic pH values (pH below the isoelectric point), protonation of surface hydroxyl groups occurs, leading to the formation of –FeOH_2_^+^ species. Conversely, at alkaline pH values (pH above the isoelectric point), deprotonation of surface hydroxyl groups results in the formation of –FeO^−^ groups, imparting a negative surface charge to the nanoparticles [28]. Consistent with this behavior, MNPs-1 exhibited a transition from positive to negative ζ-potential across their isoelectric point, located at approximately pH = 7. In contrast, MNPs-2 maintained a positive ζ-potential over the entire investigated pH range, highlighting the effect of the preparation conditions (Table 1) on this parameter. However, the ζ-potential of MNPs-2 became very low at pH 8 and above, which probably also led to the drastic increase in apparent size. DLS measurements of bare MNPs-1 and MNPs-2 revealed a hydrodynamic diameter of 764 ± 93 nm (PdI: 0.28 ± 0.05) and 34 ± 1 (PdI: 0.20 ± 0.01), respectively, when dispersed in an aqueous solution at pH 4.5, corresponding to the conditions used for enzyme immobilization (Figure 2).

**Figure 3:**
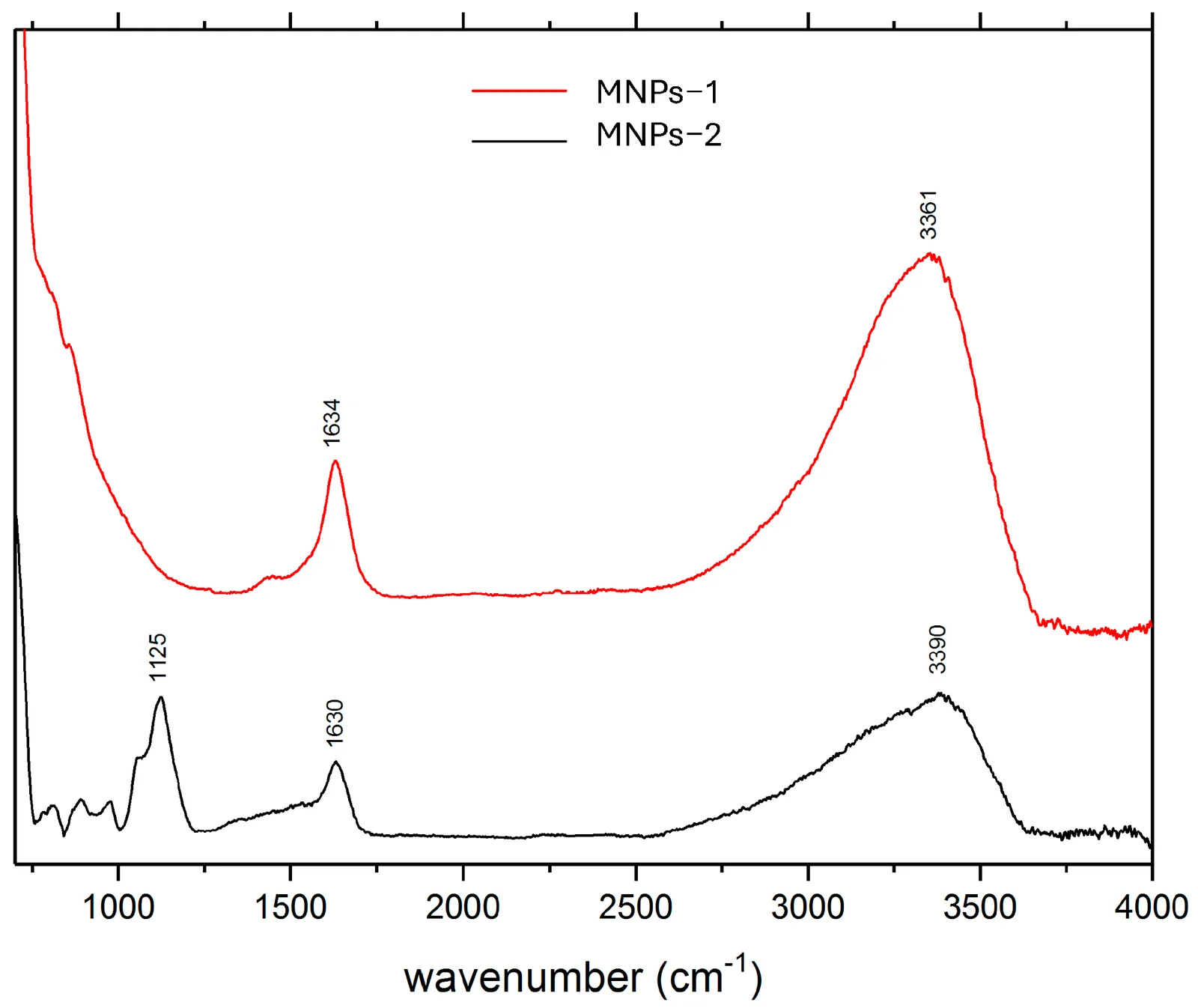
Comparison of FT-IR spectra of bare MNPs-1 and MNPs-2.

Bare MNPs-1 and MNPs-2 were characterized by FT-IR spectroscopy (Figure 3). In both spectra, the broad band observed in the 3000–3500 cm^−1^ region is attributed to O–H stretching vibrations, while the band at approximately 1630 cm^−1^ corresponds to the bending vibration of adsorbed water molecules. In the FT-IR spectrum of MNPs-1, an additional band at 1125 cm^−1^ is likely associated with residual NH_3_ originating from Mohr’s salt used for the synthesis [18,29].

### 3.2. Laccase Immobilization and Catalytic Activity in the Presence of Reducing Substrates

After synthesis and physicochemical characterization, the MNPs were evaluated as supports for laccase immobilization. The immobilization results (Table 2) show high yields for both covalent binding and adsorption approaches; however, laccase immobilized on MNPs-2 by adsorption exhibited a higher activity recovery (84%). This enhanced performance can be mainly attributed to the good dispersion of the nanoparticles and their larger specific surface area, which provide a greater number of accessible immobilization sites. Furthermore, adsorption immobilization relies on weak physical interactions rather than covalent bonds. However, despite the higher initial activity recovery, the adsorbed enzyme in MNPs-2 exhibited lower operational stability upon repeated use, likely due to partial enzyme desorption from the support and the weaker nature of the enzyme–support interactions (Figure S1) [30].

**Figure 4:**
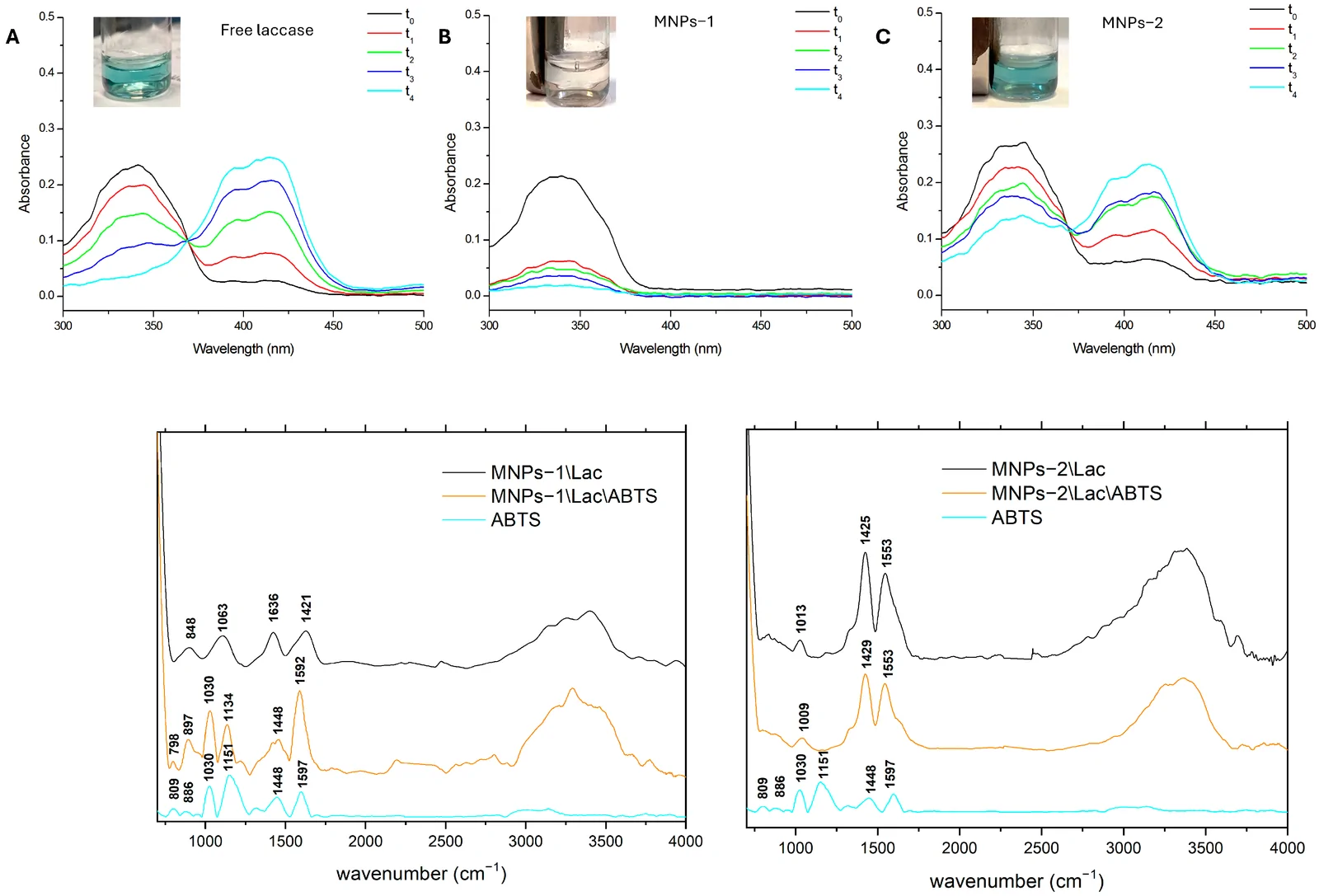
Top: Time-course UV-Vis spectra of the ABTS oxidation catalyzed by free laccase (**A**), laccase immobilized on MNPs-1 (**B**), and laccase immobilized on MNPs-2 (**C**). Spectra were recorded at different reaction times: t_0_ (reaction start), t_1_ (5 min), t_2_ (10 min), t_3_ (20 min), and t_4_ (30 min). For each system, a photograph of the reaction solution at the end of the experiment is shown. Bottom: FT-IR spectra of laccase-functionalized MNPs recorded before (black line) and after (brown line) the reaction with ABTS. The FT-IR spectrum of ABTS (light blue line) is reported as a reference [32]. Spectra of MNPs-1 are shown on the left, while those of MNPs-2 are shown on the right.

After laccase immobilization, tests with ABTS, the most commonly employed chromogenic substrate for monitoring laccase activity, were performed using MNPs-1 and MNPs-2. ABTS (reaction scheme reported in the Supplementary Materials, Scheme S1-1) was initially selected as the model substrate since it revealed markedly different catalytic behaviors depending on the supports obtained by the two different synthetic routes. ABTS is a colorless reducing substrate, and its oxidation by laccase typically leads to the formation of the green ABTS•^+^ cation radical (*E*^0^ = 0.68 V), characterized by a diagnostic absorption band at 420 nm (Figure 4A, top) [31]. The radical species is relatively stable. At room temperature in aqueous solution/buffer, it maintains a usable absorbance for approximately 12–24 h. The reaction can also proceed with a further oxidation, with the formation of the ABTS^2+^ (*E*^0^ = 1.09 V) species. In the absence of another additional substrate, fragmentation products are formed [31]. Surprisingly, in the case of laccase immobilized on MNPs-1, the expected green coloration was not observed. Instead, the reaction solution remained colorless, and only a progressive decrease in the ABTS absorption band at 340 nm was detected (Figure 4B). In contrast, laccase immobilized on MNPs-2 exhibited the typical behavior, with rapid formation of the ABTS•^+^ cation radical and the corresponding appearance of the absorption band at 420 nm (Figure 4C). Analysis of the absorbance decay at 340 nm in Figure 4A–C shows a faster consumption of ABTS for laccase immobilized on MNPs-1 compared to both laccase immobilized on MNPs-2 and the free enzyme, suggesting enhanced catalytic turnover under identical reaction conditions. However, the absence of the characteristic ABTS•^+^ signal at 420 nm indicates that the oxidation product is not detectable in solution, prompting further investigation into the fate of the radical species and their possible interaction with the nanoparticle surface.

To investigate this effect, the FT-IR spectra of laccase–MNPs-1 and laccase–MNPs-2 after reaction with ABTS were collected and compared with the IR spectrum of ABTS (Figure 4, bottom). The presence of ABTS on the surface of MNPs-1 was clearly confirmed by FT-IR analysis. In particular, the spectrum of the laccase–MNPs-1 system after reaction showed characteristic bands at 886, 1030, and 1151 cm^−1^, which can be assigned to the stretching and bending vibrations of the sulfonate groups of ABTS. In contrast, these bands were not detected in the spectra of laccase–MNPs-2 under the same conditions (see brown lines in the left and right panels of Figure 4, bottom), indicating a markedly different interaction between the reaction products and the two nanoparticle surfaces.

**Figure 5:**
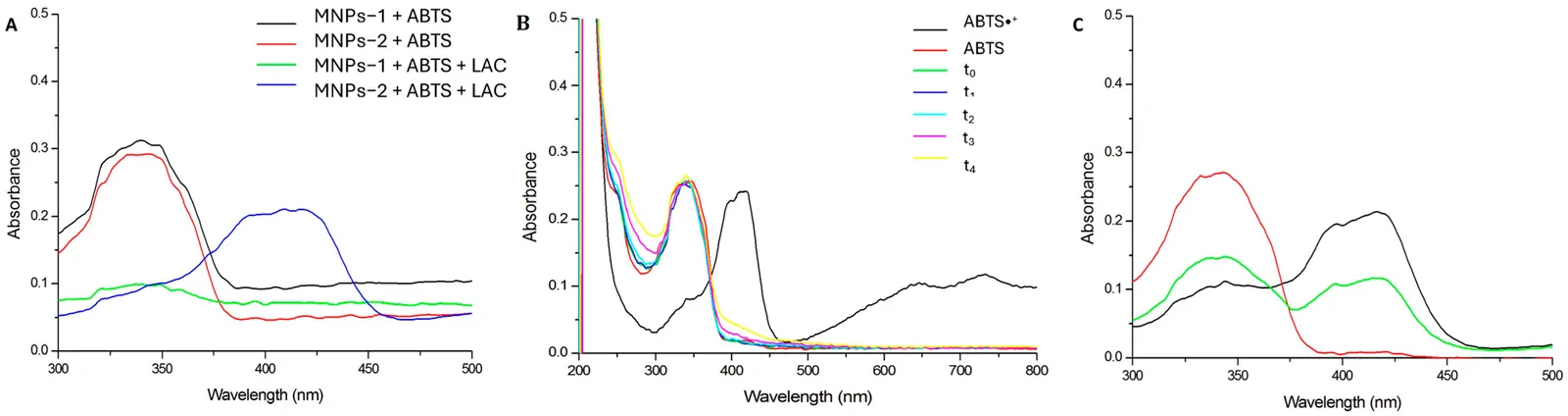
(**A**) UV-Vis spectra recorded for the reaction between bare MNPs-1 or MNPs-2 and ABTS. Black and red lines correspond to spectra of the solutions collected after 30 min of incubation of ABTS with bare MNPs-1 and MNPs-2, respectively. Green and blue lines correspond to spectra of the same solutions after the subsequent addition of free laccase. (**B**) Reaction between the ABTS•^+^ radical and Mohr’s salt (Fe^2+^). The spectra of reduced ABTS (red line) and ABTS•^+^ generated by free laccase (black line) are reported. The reaction between ABTS•^+^ and Fe^2+^ was monitored over time: t_0_ (initial time), t_1_ (5 min), t_2_ (10 min), t_3_ (20 min), and t_4_ (30 min). (**C**) Reaction of laccase immobilized on MNPs-1 with ABTS. The UV–Vis spectra of ABTS (red line), ABTS•^+^ generated by free laccase (black line), and ABTS•^+^ produced by laccase immobilized on MNPs-1 prepared with a Fe^2+^:Fe^3+^ molar ratio of 1:2 (green line) are shown.

To elucidate the mechanism underlying this unusual behavior, the interaction between the magnetic nanoparticle surface and ABTS was first investigated in the absence of laccase. Since laccase is required to promote substrate oxidation and radical formation, no reaction was expected. Accordingly, no spectral changes were observed for either MNPs-1 or MNPs-2 in the presence of ABTS alone, as evidenced by the unchanged UV–Vis spectra (Figure 5A, black and red lines). Upon subsequent addition of laccase, the characteristic ABTS•^+^ signal was readily detected for MNPs-2, whereas in the case of MNPs-1, a progressive decrease in the ABTS absorption band was observed together with the disappearance of the ABTS•^+^ signal. These results suggest that the anomalous behavior observed for laccase immobilized on MNPs-1 is not related to the reduced ABTS substrate, but specifically to the ABTS•^+^ radical species. Interaction with the nanoparticle surface occurs only in the presence of laccase, which is required to generate the radical intermediate. The distinct surface composition of MNPs-1, particularly a slight excess of Fe^2+^ ions in the outermost layer, is, therefore, proposed to be responsible for the different reactivity of the two nanoparticle systems toward ABTS•^+^. This might be consistent with the well-known influence of metal ions on laccase activity. In particular, metal ion species such as iron can contribute to enhancing the catalytic performance of laccase, as reported in the literature [33,34].

To evaluate whether Fe^2+^ could directly reduce the ABTS•^+^ radical back to its reduced form, ABTS•^+^ was first generated using free laccase and subsequently reacted with Fe^2+^ (Mohr’s salt). No spectral variations were detected, indicating that Fe^2+^ alone is not capable of reducing ABTS•^+^ under the investigated conditions (Figure 5B). To further verify the role of Fe^2+^ excess, MNPs were synthesized using a stoichiometric Fe^2+^/Fe^3+^ ratio of 1:2, avoiding any Fe^2+^ excess. As shown in Figure 5C (green line), laccase immobilized on these nanoparticles exhibited the typical ABTS oxidation product. These findings confirm that a small excess of Fe^2+^ at the nanoparticle surface plays a key role in modulating the interaction between the laccase-generated radical species and the magnetic support.

**Figure 6:**
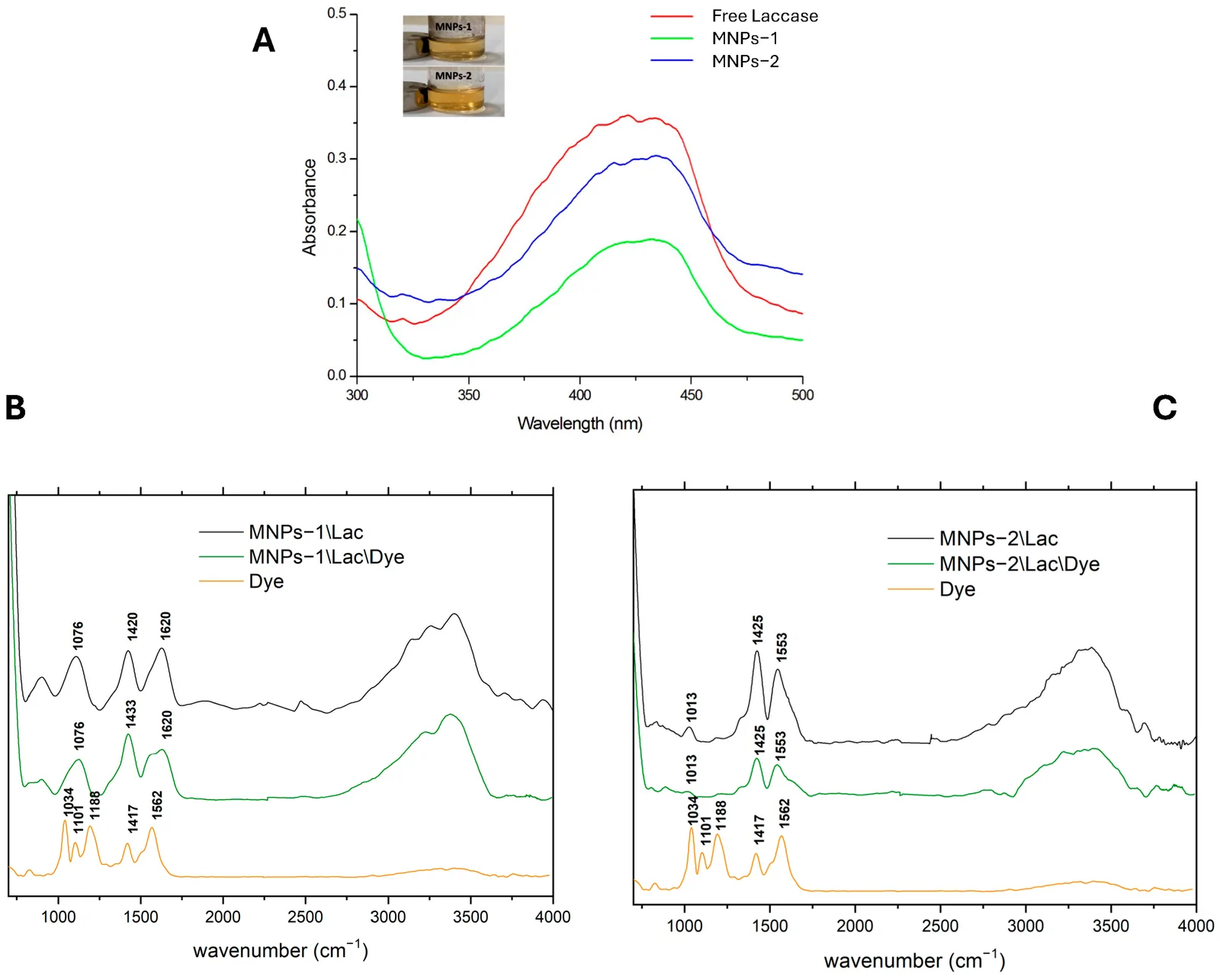
(**A**) UV-Vis spectra of the phenoxazinone dye formed from the reaction between 3-amino-4-hydroxybenzene sulfonic acid and free laccase (red line), laccase immobilized on MNPs-1 (green line), and laccase immobilized on MNPs-2 (blue line). The inset photograph shows the formation of the yellow-colored dye in the presence of laccase immobilized on both MNPs-1 and MNPs-2. (**B**,**C**) FT-IR spectra of laccase-functionalized MNPs recorded before (black line) and after (green line) the reaction with 3-amino-4-hydroxybenzene sulfonic acid. The FT-IR spectrum of the colored reaction product, 2-amino-3-oxo-3H-phenoxazine-8-sulfonic acid, is reported as a reference (orange line). The reference compound was synthesized using free laccase and 3-amino-4-hydroxybenzene sulfonic acid in an acetate buffer at room temperature. Spectra shown in panel (**B**) correspond to MNPs-1, while those in panel (**C**) correspond to MNPs-2.

These results prompted a deeper comparison of the two nanoparticle synthetic routes and an evaluation of their behavior with different reducing substrates in order to assess the suitability of the resulting MNPs as solid supports for oxidoreductase immobilization. In this context, laccases, particularly those of fungal origin, represent attractive biocatalysts, as they can promote the synthesis of novel organic compounds through both heteromolecular and homomolecular transformations [35]. Compounds with phenazine or a phenoxazine structures can be easily obtained through biocatalysis using amino phenolic precursors [21,36,37,38]. The reactivity of laccase immobilized on MNPs-1 and MNPs-2 was tested with 3-amino-4-hydroxybenzene sulfonic acid. The homocoupling reaction leads to the formation of a colored compound with a phenoxazinone structure through the Michael reaction (Scheme S1) [21]. The radical reaction proceeds fast toward the formation of the dye with the appearance of the absorbance band at 420 nm, as shown in Figure 6A, where the synthesis of the dye, obtained by the use of MNPs-1 and MNPs-2, is compared with that obtained by the use of free laccase. The solution is colored, and in both cases, the FT-IR analysis confirms the absence of the dye on the surface of the nanoparticles, since after reaction with the substrate, the surface remains unmodified (see Figure 6B,C).

The absence of interaction with the MNP surface may be attributed to the fast homocoupling reaction of the substrate radical species once formed and the non-radical character of the obtained product.

**Figure 7:**
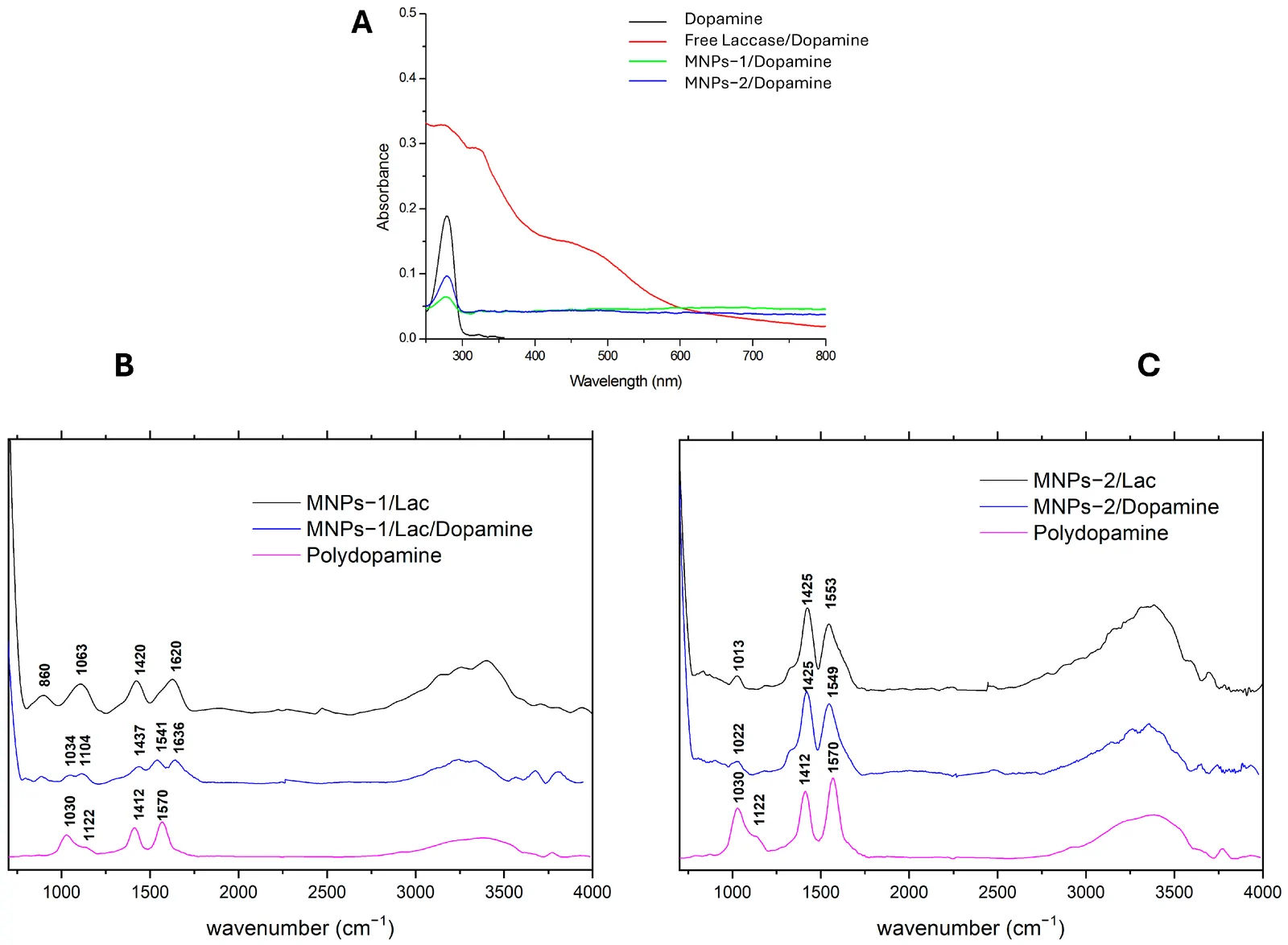
(**A**) UV-Vis spectra of the reaction solutions obtained after the oxidation of dopamine catalyzed by free laccase (red line), laccase immobilized on MNPs-1 (green line), and laccase immobilized on MNPs-2 (blue line). The UV–Vis spectrum of dopamine is shown as a reference (black line). (**B**,**C**) FT-IR spectra of laccase-functionalized MNPs recorded before (black line) and after (blue line) the reaction with dopamine. The FT-IR spectrum of polydopamine (purple line), synthesized using free laccase and dopamine in acetate buffer at room temperature, is reported as a reference. Spectra corresponding to MNPs-1 are shown on the left (**B**), while those corresponding to MNPs-2 are shown on the right (**C**).

Laccases are also capable of catalyzing polymerization reactions starting from catechol monomers. Dopamine (DA), a catecholaminergic neurotransmitter, can adhere to various substrates through self-polymerization, forming polydopamine (PDA) (Scheme S1-(3) [39]. In this study, polydopamine synthesis was performed to evaluate the activity of laccase immobilized on MNPs-1 and MNPs-2. Figure 7A (red line) shows the UV–Vis spectrum of polydopamine synthesized using free laccase. The spectrum is characteristic of melanin-like pigments, with a gradual increase in absorption in the UV region. Broad absorption bands from 200 to 500 nm are observed, which can be attributed to the formation of 5,6-dihydroxyindole units [40,41]. In contrast, when dopamine is reacted with laccase immobilized on MNPs-1 or MNPs-2, only a decrease in the dopamine peak at 290 nm is observed (Figure 7A, green and blue lines). The spectrum of unreacted dopamine is shown for comparison (Figure 7A, black line). To verify whether polydopamine is chemisorbed on the MNPs, IR spectra were recorded and are presented in Figure 7B,C.

These results indicate that PDA is deposited on the MNP surface via dopamine polymerization catalyzed by the oxidizing activity of the immobilized laccase, proceeding through a radical-mediated process. While dopamine self-polymerization at alkaline pH on MNPs is well documented, in this study, the coating occurs under acidic conditions. This may offer an advantage for applications requiring acidic media; for example, when coating is performed in the presence of acid-soluble biopolymers, such as chitosan [39].

## 4. Conclusions

This study provides new insights into the behavior of immobilized laccase and highlights the critical role played by the physicochemical properties of magnetic nanoparticle supports. While enzyme immobilization is widely used to enhance catalyst stability and reusability, the present results demonstrate that, for oxidative enzymes such as laccases, the interaction between the enzyme-generated radical species and the support surface is a key factor governing the overall catalytic outcome. Laccase immobilized on magnetite nanoparticles synthesized under different conditions exhibited markedly different catalytic behaviors. In particular, MNPs-1, characterized by larger aggregates and an excess of surface-exposed Fe^2+^ ions, strongly interacted with radical intermediates generated during laccase-catalyzed oxidation reactions. This interaction led to the adsorption or surface confinement of reaction products, as observed for ABTS•^+^ and polydopamine formation, and resulted in an apparent increase in substrate consumption. However, this behavior also altered the expected reaction pathway and limited product recovery in solution, indicating that the nanoparticle surface actively participates in the redox process rather than acting as an inert support. In contrast, laccase immobilized on MNPs-2, which displays smaller particle size, improved dispersion, and a more controlled Fe^2+^/Fe^3+^ surface composition, showed catalytic behavior closely resembling that of the free enzyme. Radical products were formed and released into solution without significant interaction with the support, confirming that these nanoparticles provide a more suitable environment for laccase-mediated oxidative transformations due to the smaller size, reduced aggregation state, and less detrimental interaction with the substrate. The observed differences were consistent across multiple substrates, including chromogenic probes and phenolic monomers, demonstrating that the nature of the radical species and their stability critically influence their interaction with the support. These findings emphasize that laccase activity, selectivity, and apparent efficiency cannot be evaluated independently of the support surface chemistry, especially when radical-mediated mechanisms are involved. Overall, this work underscores the importance of tailoring magnetic nanoparticle properties to the specific requirements of laccase-catalyzed reactions. Controlling nanoparticle synthesis parameters, particularly those affecting surface Fe^2+^ availability and colloidal stability, is essential to preserve the intrinsic catalytic behavior of laccase and to avoid undesired surface-driven side effects. On the other hand, these results can provide an easy procedure to get a reactive coating of magnetic nanoparticles and a valuable guideline for the rational design of laccase-based magnetic biocatalysts for oxidative and synthetic applications.

## Figures and Tables

**Table 1:** Comparison between the MNPs-1 and MNPs-2 synthesis.

Parameters	MNPs-1	MNPs-2
Temperature	60 °C	25 °C
Fe^2+^ salt	Fe(NH_4_)_2_(SO_4_)_2_·6H_2_O	FeCl_2_
Fe^3+^ salt	FeCl_3_·6H_2_O	FeCl_3_
Fe^2+^/Fe^3+^ molar ratio	1.1:2	1:2
Precipitant agent	NaOH	NH_4_OH
N_2_ gas	Yes	Yes

**Table 2:** Immobilization yield and activity recovery of laccase covalently immobilized on MNPs-1 and absorbed on MNPs-1 and MNPs-2 using 3-amino-4-hydroxybenzenesulfonic acid as a substrate.

	Immobilization Yield (%)	Activity Recovery (%)
MNPs-1 (Covalent immobilization)	81 ± 6	53 ± 10
MNPs-1 (Adsorption immobilization)	98 ± 1	54 ± 5
MNPs-2 (Adsorption immobilization)	98 ± 0.5	84 ± 4

## Data Availability

The original contributions presented in this study are included in the article/Supplementary Materials. Further inquiries can be directed to the corresponding authors.

## Supplementary Materials

The following supporting information can be downloaded at: https://www.mdpi.com/article/10.3390/biom16091244/s1. Scheme S1. Laccase reactions with different reducing substrates: (1) 2,2’-azino-bis (3-ethylbenthiazoline-6-sulphonic acid) (ABTS), (2) 3-amino-4-hydroxybenzene sulfonic acid and (3) dopamine. The reactions were carried out in acetate buffer pH = 4.5 at room temperature. Figure S1. Reusability of laccase immobilized on MNPs-1 via covalent and adsorption methods (blue and violet lines, respectively) and on MNPs-2 via adsorption (red line).

## Abbreviations

The following abbreviations are used in this manuscript:

MNPs	Magnetic nanoparticles
GRAS	Generally recognized as safe
APTES	(3-aminopropyl) triethoxysilane
ABTS	2,2′-azino-bis(3-ethylbenzothiazoline-6-sulfonic acid)
DDW	Double distilled water
AFM	Atomic force microscopy
TEM	Transmission electron microscopy
FT-IR	Fourier-transform infrared spectroscopy
*Tv*	*Trametes versicolor*
PBS	Phosphate buffer
A_f_	Free laccase activity
A_wb_	Laccase activity in washing buffer
A_im_	Activity of immobilized enzyme
DLS	Dynamic light scattering
DA	Dopamine
PDA	Polydopamine
XRD	X-ray diffraction
XPS	X-ray photoelectron spectroscopy

## References

[1] Wang H., Tang L.X., Ye Y.F., Ma J.X., Li X., Si J., Cui B.K. (2024). Laccase Immobilization and Its Degradation of Emerging Pollutants: A Comprehensive Review. J. Environ. Manag..

[2] Sheldon R.A., Woodley J.M. (2018). Role of Biocatalysis in Sustainable Chemistry. Chem. Rev..

[3] Cherry J.R., Fidantsef A.L. (2003). Directed Evolution of Industrial Enzymes: An Update. Curr. Opin. Biotechnol..

[4] Chapman J., Ismail A.E., Dinu C.Z. (2018). Industrial Applications of Enzymes: Recent Advances, Techniques, and Outlooks. Catalysts.

[5] Zhou C., Dong A., Wang Q., Yu Y., Fan X., Cao Y., Li T. (2017). Effect of Common Metal Ions and Anions on Laccase Catalysis of Guaiacol and Lignocellulosic Fiber. BioResources.

[6] Sheldon R.A., van Pelt S. (2013). Enzyme Immobilisation in Biocatalysis: Why, What and How. Chem. Soc. Rev..

[7] Basso A., Serban S. (2019). Industrial Applications of Immobilized Enzymes—A Review. Mol. Catal..

[8] Di Cosimo R., Mc Auliffe J., Poulose A.J., Bohlmann G. (2013). Industrial Use of Immobilized Enzymes. Chem. Soc. Rev..

[9] Zdarta J., Meyer A.S., Jesionowski T., Pinelo M. (2018). A General Overview of Support Materials for Enzyme Immobilization: Characteristics, Properties, Practical Utility. Catalysts.

[10] Bilal M., Zhao Y., Rasheed T., Iqbal H.M.N. (2018). Magnetic Nanoparticles as Versatile Carriers for Enzymes Immobilization: A Review. Int. J. Biol. Macromol..

[11] Markides H., Rotherham M., El Haj A.J. (2012). Biocompatibility and Toxicity of Magnetic Nanoparticles in Regenerative Medicine. J. Nanomater..

[12] He G., Liu H., Yang C., Hu K., Zhai X., Fang B., Liu K., Zulekha, Li D. (2024). A Comparison of Dual-Enzyme Immobilization by Magnetic Nanoparticles and Magnetic Enzyme Aggregates for Cascade Enzyme Reactions. Biochem. Eng. J..

[13] Kidibule P.E., Costa J., Atrei A., Plou F.J., Fernandez-Lobato M., Pogni R. (2021). Production and Characterization of Chitooligosaccharides by the Fungal Chitinase Chit42 Immobilized on Magnetic Nanoparticles and Chitosan Beads: Selectivity, Specificity and Improved Operational Utility. RSC Adv..

[14] Wang B., Bian C., Lu X., Ma Y., Li N., Xiong D., Xu X., Yang Z., Zhang H., Guan T. (2025). Laccase Immobilized on Magnetic Nanoparticles for the Degradation of Imidacloprid: Based on Multi-Spectroscopy and Molecular Docking. Spectrochim. Acta A Mol. Biomol. Spectrosc..

[15] Sharma N.D. (2025). Reaction Mechanism and Redox Potential of Laccase. Laccase and Polyphenol Oxidase: Biochemistry and Biotechnological Applications.

[16] Pomogailo S.I., Chepaikin E.G., Bubelo O.N., Jussupkaliyeva R.I., Kustov L.M. (2024). Magnetic Nanocomposites Based on Iron Oxides as Catalysts of Oxidation Reactions. Crystals.

[17] Atrei A., Mahdizadeh F.F., Baratto M.C., Scala A. (2021). Effect of Citrate on the Size and the Magnetic Properties of Primary Fe3o4 Nanoparticles and Their Aggregates. Appl. Sci..

[18] Atrei A., Lesiak-Orlowska B., Tóth J. (2022). Magnetite nanoparticles functionalized with citrate: A surface science study by XPS and ToF-SIMS. Appl. Surf. Sci..

[19] De Sousa M.E., Fernández Van Raap M.B., Rivas P.C., Mendoza Zélis P., Girardin P., Pasquevich G.A., Alessandrini J.L., Muraca D., Sánchez F.H. (2013). Stability and Relaxation Mechanisms of Citric Acid Coated Magnetite Nanoparticles for Magnetic Hyperthermia. J. Phys. Chem. C.

[20] Feng J., Yu S., Li J., Mo T., Li P. (2016). Enhancement of the Catalytic Activity and Stability of Immobilized Aminoacylase Using Modified Magnetic Fe3O4 Nanoparticles. Chem. Eng. J..

[21] Forte S., Polak J., Valensin D., Taddei M., Basosi R., Vanhulle S., Jarosz-Wilkolazka A., Pogni R. (2010). Synthesis and Structural Characterization of a Novel Phenoxazinone Dye by Use of a Fungal Laccase. J. Mol. Catal. B Enzym..

[22] Saragi T., Depi B.L., Butarbutar S., Permana B., Risdiana (2018). The Impact of Synthesis Temperature on Magnetite Nanoparticles Size Synthesized by Co-Precipitation Method. J. Phys. Conf. Ser..

[23] Forge D., Roch A., Laurent S., Tellez H., Gossuin Y., Renaux F., Elst L.V., Muller R.N. (2008). Optimization of the Synthesis of Superparamagnetic Contrast Agents by the Design of Experiments Method. J. Phys. Chem. C.

[24] Schwaminger S.P., Syhr C., Berensmeier S. (2020). Controlled Synthesis of Magnetic Iron Oxide Nanoparticles: Magnetite or Maghemite?. Crystals.

[25] Maity D., Agrawal D.C. (2007). Synthesis of Iron Oxide Nanoparticles under Oxidizing Environment and Their Stabilization in Aqueous and Non-Aqueous Media. J. Magn. Magn. Mater..

[26] Alibeigi S., Vaezi M.R. (2008). Phase Transformation of Iron Oxide Nanoparticles by Varying the Molar Ratio of Fe2+:Fe3+. Chem. Eng. Technol..

[27] Meng J.H., Yang G.Q., Yan L.M., Wang X.Y. (2005). Synthesis and Characterization of Magnetic Nanometer Pigment Fe3O4. Dye. Pigment..

[28] Zhang J., Lin S., Meiling H., Qing S., Liancu X. (2020). Adsorption Properties of Magnetic Magnetite. Water.

[29] Jain R., Gulati S. (2023). Influence of Fe2+ Substitution on FTIR and Raman Spectra of Mn Ferrite Nanoparticles. Vib. Spectrosc..

[30] Zucca P., Sanjust E. (2014). Inorganic Materials as Supports for Covalent Enzyme Immobilization: Methods and Mechanisms. Molecules.

[31] Brogioni B., Biglino D., Sinicropi A., Reijerse E.J., Giardina P., Sannia G., Lubitz W., Basosi R., Pogni R. (2008). Characterization of Radical Intermediates in Laccase-Mediator Systems. a Multifrequency EPR, ENDOR and DFT/PCM Investigation. Phys. Chem. Chem. Phys..

[32] Huang J., Yang Y., Wang Y., Zhang M., Liu Y. (2018). Immobilization of a Laccase/2,2′-Azino-Bis-(3-Ethylbenzothiazoline)-6-Sulfonic Acid System to Layered Double Hydroxide/Alginate Biohybrid Beads for Biodegradation of Malachite Green Dye. BioMed Res. Int..

[33] Othman A.M., Flaifil A.G. (2025). Characterization and Evaluation of the Immobilized Laccase Enzyme Potential in Dye Degradation via One Factor and Response Surface Methodology Approaches. Sci. Rep..

[34] Muthuvelu K.S., Rajarathinam R., Selvaraj R.N., Rajendren V.B. (2020). A Novel Method for Improving Laccase Activity by Immobilization onto Copper Ferrite Nanoparticles for Lignin Degradation. Int. J. Biol. Macromol..

[35] Mikolasch A., Schauer F. (2009). Fungal Laccases as Tools for the Synthesis of New Hybrid Molecules and Biomaterials. Appl. Microbiol. Biotechnol..

[36] Sousa A.C., Conceição Oliveira M., Martins L.O., Robalo M.P. (2018). A Sustainable Synthesis of Asymmetric Phenazines and Phenoxazinones Mediated by CotA-Laccase. Adv. Synth. Catal..

[37] Sousa A.C., Oliveira M.C., Martins L.O., Robalo M.P. (2014). Towards the Rational Biosynthesis of Substituted Phenazines and Phenoxazinones by Laccases. Green Chem..

[38] Wlizło K., Polak J., Jarosz-Wilkołazka A., Pogni R., Petricci E. (2020). Novel Textile Dye Obtained through Transformation of 2-Amino-3-Methoxybenzoic Acid by Free and Immobilised Laccase from a Pleurotus Ostreatus Strain. Enzym. Microb. Technol..

[39] Costa J., Baratto M.C., Spinelli D., Leone G., Magnani A., Pogni R. (2024). A Novel Bio-Adhesive Based on Chitosan-Polydopamine-Xanthan Gum for Glass, Cardboard and Textile Commodities. Polymers.

[40] Zou Y., Chen X., Yang P., Liang G., Yang Y., Gu Z., Li Y. (2020). Regulating the Absorption Spectrum of Polydopamine. Sci. Adv..

[41] Deng M., Zhao H., Zhang S., Tian C., Zhang D., Du P., Liu C., Cao H., Li H. (2015). High Catalytic Activity of Immobilized Laccase on Core–Shell Magnetic Nanoparticles by Dopamine Self-Polymerization. J. Mol. Catal. B Enzym..

