# Supplementary material for: Unveiling the Physicochemical Properties of Magnetic Nanoparticles as Solid Carriers for Laccase Immobilization Toward Different Reducing Substrates
Source: Biomolecules. 2026 Aug 27;16(9):1244. doi: 10.3390/biom16091244 (PMC13604739; doi:10.3390/biom16091244)
Supplement: Supplementary file 1 [file biomolecules-16-01244-s001.zip › biomolecules-4480480-supplementary.pdf]

Supplementary Materials

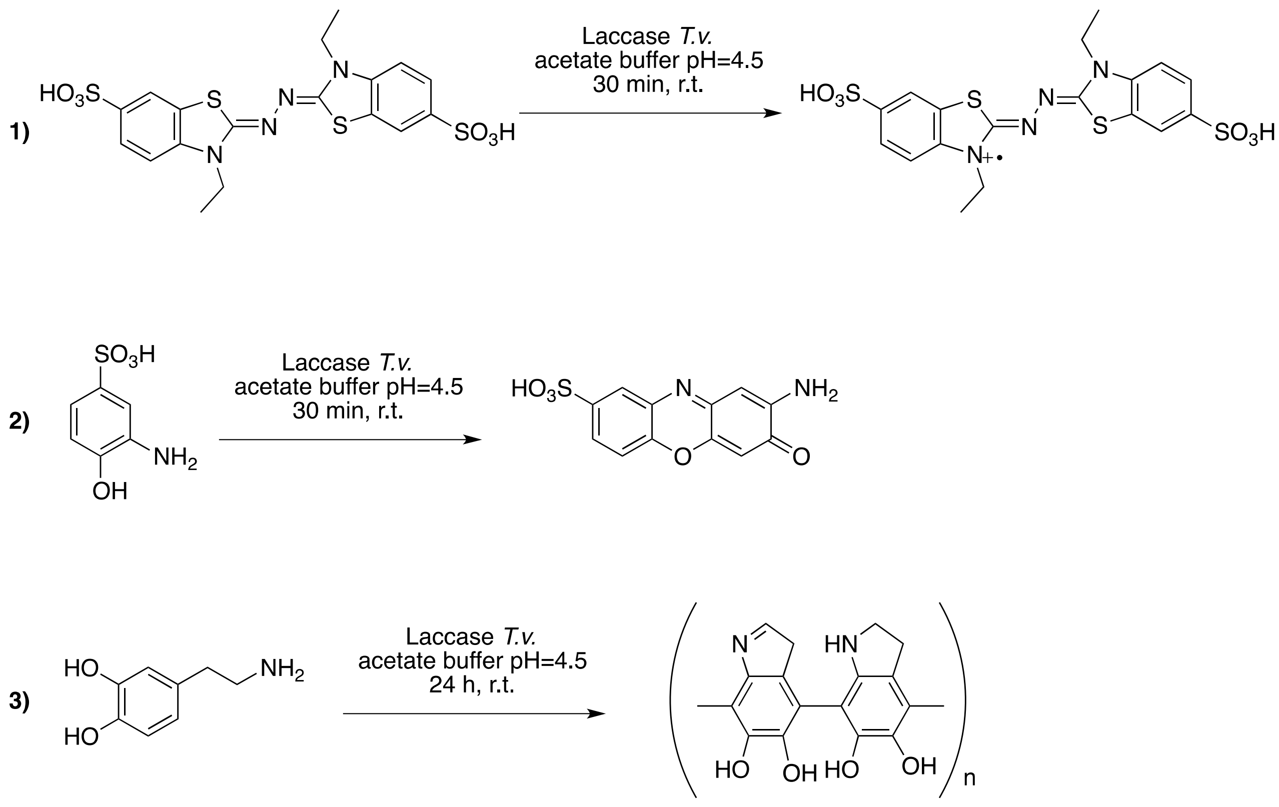

Scheme S1. Laccase reactions with different reducing substrates: 1) 2,2′-azino-bis (3-ethylbenthiazoline-6-sulphonic acid) (ABTS), 2) 3-amino-4-hydroxybenzene sulfonic acid and 3) dopamine. The reactions were carried out in acetate buffer pH= 4.5 at room temperature.

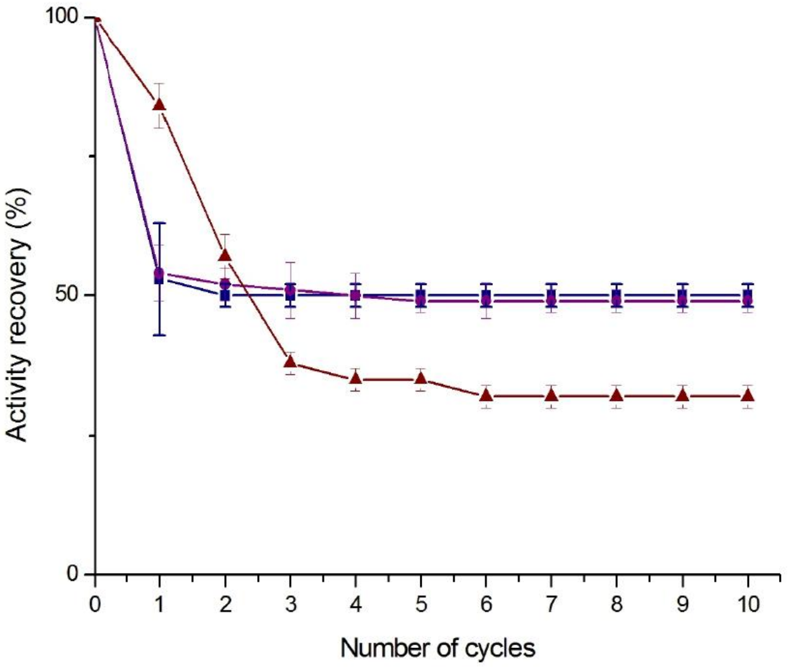

Figure S1. Reusability of laccase immobilized on MNPs-1 via covalent and adsorption methods (blue and violet lines, respectively) and on MNPs-2 via adsorption (red line).
